# Role of Communication Strategies in Organizational Commitment, Mediating Role of Faculty Engagement: Evidence From English Language Teachers

**DOI:** 10.3389/fpsyg.2022.921797

**Published:** 2022-06-22

**Authors:** Yan Ma

**Affiliations:** School of Foreign Languages, Lanzhou City University, Lanzhou, China

**Keywords:** information flow, information adequacy, information feedback, faculty engagement, organizational commitment

## Abstract

Employees are critical stakeholders for an organization because they directly deal with the end-users and represent the entire firm. To recognize the strategic importance of the employees, organizations create communication programs to keep employees apprised of organizational issues. In this regard, this study examined the role of communication strategies (i.e., information flow, information adequacy, and information feedback) on organizational commitment. The study also investigated the mediating effect of faculty engagement between communication strategies and organizational commitment. Self-administered survey aided in acquiring data from 276 English language teachers in China. The analysis of the data was conducted using SmartPLS through the Structured Equation Modeling technique. The outcome of the study demonstrated that information flow and information feedback significantly impact organizational commitment and faculty engagement. The analysis also revealed that information adequacy significantly impacts organizational commitment but has no relationship with faculty engagement. The mediation analysis demonstrated that faculty engagement mediated the relationship between information flow and organizational commitment and between information feedback and organization commitment. However, faculty engagement did not mediate the relationship between information adequacy and organizational commitment among English language teachers in China. In theoretical terms, the study contributed in terms of incorporating different communication strategies and examining their effect on organizational commitment and faculty engagement. In practical terms, this study would be beneficial for the management of the educational institutes to develop different ways of enhancing communication strategies within the institute. This study also provided directions for the future, for example, conducting the study on other subject teachers, increasing the sample, carrying out the research in a different context, and adding different mediators and moderators in the existing model.

## Introduction

Faculty members have several important obligations toward their organizations. They are important stakeholders of organizations due to their professional positions and capacity to impact outsiders (Kim and Rhee, 2011). Further, they noticed that educational institutions now realize the strategic importance of teachers for enhancing the performance of the institution. Therefore, these organizations develop systematic communication programs to keep them informed about the organizational issues. These communication programs consist of managers, supervisors, administrators, and internal communication teams (Rhee and Moon, 2009). The importance of faculty members is quite similar to employees of any organization. So, when organizations provide effective internal communication to employees about the organizational goals, they are more likely to work hard. Such employees advocate for the organization to everyone else and establish a strong relationship with the organization (Mishra et al., 2014).

Faculty members are the most valuable asset of an institution because they have an important role in maintaining sustainability. Their functionality is varied across different institutions. Therefore, it becomes difficult to manage their roles appropriately. Some faculty members are more devoted, and they try to deliver more than what is required by their employer organizations. This devotion of faculty members enhances their work engagement which defines the significance of their roles in academic performance. So, why do some faculty members get more involved in the institution's operations? In the literature on organizational behavior and management, “organizational commitment” seems widely used. According to a research, faculty opinions toward their institution significantly impact their commitment (Jing and Zhang, 2014). Organizational commitment is a behavioral disposition that refers to an individual's willingness to remain employed. It motivates the employees to become high performers. It also leads to put up maximum effort in the company's best interests (Lovakov, 2016). According to studies, one of the most important determinants of the additional activities of faculty members is organizational commitment (Lovakov, 2016). Professionals who are prominently committed to the organization consider the work in a more favorable dimension. They desire to stay employed in the company, and are much more satisfied with their work. It is assumed that a committed employee is a good fighter for the organization (Lawrence et al., 2012). There are many factors which help in strengthening the commitment of employees with organization. Effective communication is one of these factors at organizational level.

Professional communication is acknowledged as a subset of organizational communication. It is vital for shaping employee-organization ties (in this case, faculty–organization interactions). It is also important for boosting workplace productivity (Men, 2014). The relationship development initiatives at organizational level such as communication efforts may result in two critical outcomes. These include job engagement and organizational commitment (Mishra et al., 2014). Employee engagement (faculty engagement in this study) is better described as a pleasant and rewarding state of mind which relates to commitment, vitality, and immersion at work (Schaufeli et al., 2006). Such engagement is all about getting involved in work at hand and feeling energized by it. Based on trust and fulfillment criteria, commitment is one of the most commonly cited factors of faculty–organization relationship quality in public relations (Men, 2014). A research implies that engagement's role in faculty–organization relationships is understudied (Saks, 2019). This is a shortcoming, because job engagement has been shown to be a determinant of long-term organizational commitment in studies (Saks, 2019). It is also critical to study faculty engagement to comprehend that what may drive organizational commitment in educational institutions (Harrison et al., 2017).

Human capital having lots of energy, effectiveness, and commitment are required to achieve competitive edge in the modern world. Several organizations are well aware that engagement and commitment are crucial to their competitiveness. Faculty members also have the responsibility to keep their organizations competitive. So, they are anticipated to have high levels of professional motivation, dedication, and job engagement. Disengaged teachers are upset and unsatisfied with their employment. Therefore, they underperform and have an adverse influence on their coworkers' efforts in the organizations. On the contrary, engaged faculty members are wholly devoted, committed, and determined to their work. An engaged staff can boost an organization's competitive advantage (Harrison et al., 2017). Effective communication is one of the important tasks of all academic, profit, and non-profit organizations. A growing body of research on higher education institutions have found that communication is critical for molding work attitudes and organizational productivity. It strengthens an organization's vision, activities, and procedures. It also helps in accelerating organizational improvements (Keyton, 2017). Communication is dependent on the possibilities of interaction and relationship development. It provides stimulation for professional and personal relationships through a variety of organizational communication methods (Murphy, 2015). During the recent years, it is observed that much attention has been paid to the enthusiasm and prestige of teachers in educational institutions. On the other hand, communication attribute has been neglected. Therefore, consideration to communication is critical for higher education, both in terms of success and efficacy (Avotra et al., 2021; Yingfei et al., 2021).

Communication at the organizational level is the process by which people share information relevant to the organization strategy, activities, and processes (Samson, 2018). Lateral communication refers to communication of data at the same level. Vertical communication refers to distribution of information either upward or downward between different positions in the organization. Both types of communications are accomplished through official or unstructured communication channels. It is important to provide a platform of communication to employees and supervisors for maintaining organizational activities. It may help in effective communication between the workers operating in different departments at various levels (Hee et al., 2019). According to a researcher, communication has two components at organizational level. The first is the content or the information which is communicated. The flow of information or “how information is shared inside an organization” is the other factor to consider. The flow of communication is a facet of the broader notion of psychological climate. It refers to an employee's interpretations of communication at work (Smidts et al., 2001, p. 1059). To measure communication content in organizational communication, the categories such as access to information, information adequacy, company information, and feedback are developed (Downs and Hazen, 1977; Goldhaber and Krivonos, 1977).

The demand for interaction, confidence in seniors, and communication linkages described by Roberts and O'Reilly (1974) are concerned with how information is shared in organizations. According to Hargie (2009), the function of internal communications is shifting through one to two-way communications. One-way communication is largely concerned with regulating employee conduct to assure adherence to work. It is also associated with dissemination of the previously decided choices without participation of workers. Two-way conversation at organizational level is defined as workers' participation and involvement in decision making practice. According to Hargie (2009), one-way communication indicates an asymmetrical point of view, while two-way communication reflects a harmonious viewpoint. To summarize different levels of communication strategies, several people place a strong emphasis on the informational side of organizational communication. Some place an emphasis on the environment when it comes to workplace communication. Internal communication tactics may reflect different organizational viewpoints. In terms of communication strategies for teaching English language by the faculty, authors divided these in three elements. These strategies include information flow, information adequacy, and information feedback. Previously, role of information flow and information adequacy have been explored in general perspective of organizational commitment (Walden et al., 2017). Researchers did not explore the impact of these communication strategies along with the feedback of information on faculty engagement and organizational commitment.

To fill this gap, authors tried to explore the impact of information flow, information adequacy, and information feedback suggested by Walden et al. (2017) on faculty engagement leading to the organizational commitment in educational institutions of English teaching. This study is based on certain questions such as what are the possible relationships between information flow, adequacy, and feedback with organizational commitment of faculty members. This study also tries to find out the mediating role of faculty engagement between these relationships instead of job or work engagement of the employees of the organizations as suggested by Walden et al. (2017). The results of this study give directions regarding internal communication strategies and their role in achieving organizational commitment of faculty members.

## Theoretical Underpinning

This study is inspired by relationship management theory by Ledingham (2003) and the public relations literature on faculty–organizational relationships. Cultural and societal roots, relationship development tactics, communications, and outcomes of interactions make up the relationships (Men, 2014). The relationships, hence developed, are the changeable outcomes of interactions between an organization and the various organizational and the outside groups (Ledingham, 2003). This seems to be true for different users and faculty–organization relationships administration, in which the organization increasingly builds and strives to keep connections with the faculty throughout the times. The conventional notion of integration of employees within organizations is related to this relationship viewpoint (Zerfass et al., 2018). The faculty relationships are more likely to be open, trusting, and credible when educational institutions implement faculty-centered communication tactics and develop an internal climate based on collaboration with instructors and staff (Zerfass et al., 2018). According to research, the quality of the faculty–organization relationship is related to the efforts of organizations to create ties with teachers and employees. The faculty perspectives on the quality of the faculty–organization connection are favorably predicted by the integration of faculty members in the organization. Furthermore, the faculty perceptions of the faculty–organization interaction are favorably associated with transformational leadership style (Men, 2014).

Information flow, interaction cooperativeness, and information adequacy are investigated as the components of internal communication and predictor variables of relationship management effects in this study. Interaction cooperativeness considers faculty getting about the content of communication on individual and organizational issues, while information adequacy considers faculty views about the content of communication on individual and organizational issues. Investigating faculty engagement as a prospective relationship result is one strategy to enhance relationship management theory in the context of faculty–organization connections (Walden et al., 2017). Faculty engagement ought to be a logical byproduct of strategic communication, according to Ruck and Welch's (2012) evaluations of the literature. Although the current empirical studies on the engagement framework consider that engagement has yet to be incorporated into the faculty–organization relationship paradigm (Mishra et al., 2014). Authors introduce faculty engagement as a particular type of engagement in the current framework to progress this theory. The relationship management theory has also previously provided basis for the relationships among employees through communication strategies Walden et al. (2017) which suggested that it could also be utilized as a foundational stone in our research. This study tries to find possible impact of communication strategies among faculty members to achieve organizational commitment.

This study also got a support from the theories of organizational commitment including behavioral commitment theory, transactional commitment theory, obligatory commitment theory, and attitudinal commitment theory (Becker, 1960). Theories that use an attitudinal concept of commitment emphasize on an individual's desire to stay in an organization. According to this hypothesis, an individual's commitment to an organization was likely influenced by emotions of cohesion or connection with that group (Meyer, 1984). With the help of this theory, it is assumed that faculty engagement is a behavioral aspect which may lead to their commitment with the organization. Therefore, this theory provides a foundational basis of organizational commitment by the faculty members.

### Communication Strategies and Faculty Engagement

According to the analysis of the literature, the level of employee engagement (faculty engagement in this case) first gained traction as a topic of substantial scientific investigation with Kahn's (1990) research. Kahn's (1990) research characterized engagement as the presentation of someone's identity *via* job behaviors which encourage commitment to work. Work engagement, on the other hand, has been the topic of discussion since Kahn (1990) stated this definition as to what it genuinely comprises. The argument includes whether work engagement differs from other ideas such as organizational satisfaction and commitment, and what exactly employees are involved with at work. “Being dedicated to an organization is different than being engaged to the activities done within it”, it is also wise to note (Vecina et al., 2013, p. 293). According to studies, there is a difference between work engagement (the subject of this study) with organizational engagement, that is, a type of organizational commitment. To distinguish the concept of job engagement from that of organizational commitment, authors employ the label job engagement of faculty in this study. Job engagement entails a task orientation as well as one's work position. Job engagement is a psychological condition which workers perceive (similar to flow and also being taken away for work), so it describes whether people engage with their employment or not (Saks, 2019). Organizational commitment, on the other hand, is an emotional tie to the organization that includes cost concerns and a social obligation to the organization (Meyer, 1984).

A prospective study has demonstrated that more engaged and determined people with their occupations have the probability to keep ties with their employers for long. It demonstrates strong organizational commitment (Lesener et al., 2019). However, there are some similarities between work engagement and organizational commitment, they are not the same. Faculty members' job engagement is defined as their engagement with activities, passion for jobs, and ability to work effectively. Engagement is made up of some of the following elements: Vigor, commitment, and assimilation. At job, vigor entails a high degree of energy and mental fortitude, a determination to put effort into one's task, and perseverance in the face of adversity (Schaufeli et al., 2006). The feeling of importance, passion, motivation, dignity, and struggle are all characteristics of commitment. When somebody is entirely attentive and pleasantly immersed in their task, assimilation happens, and time passes swiftly (Schaufeli et al., 2006). Engagement is a psychological condition which lasts longer and therefore is separate from many other conceptions such as organizational commitment (Saks, 2019). Work engagement is defined in this study as a condition of absorption in working in which faculty members show passion for accomplishing a specialized job while retaining a strong emotional attachment to their job position.

When faculty members obtain resources and support from their organization, some of them feel obligated to reciprocate it by immersing themselves deeper wholeheartedly in their roles (Saks, 2019). Faculty members can absorb themselves into the day-to-day activities and experience a sense of belongingness with this support. When faculty members believe their organization and supervisors support them, they experience work and job-related engagement (Schaufeli et al., 2006). According to Ruck and Welch (2012), organizations must meet the following six requirements to engender the behaviors associated with engagement: simply stating the employee's role in an organization, assisting the employee in identifying with the organization, making sure workers feel that they have organizational support, providing information that assists employees in understanding corporate goals and strategy.

Educational organizations should evaluate information flow, adequacy of the information, feedback of the information, and interactive support as characters of communication which are likely to lead to work engagement when interacting with staff and faculty members. Such factors measure how faculty members perceive the access of work and relevant information about the organization, and they handle the common layers of communication inside organizations (organization to faculty and manager to faculty) (Rhee and Moon, 2009). The open interchange of ideas, topics, and views through an organization's vertical and horizontal networks is referred to as information flow. Employees' perceptions of information adequacy are defined as their belief that they are obtaining the quantity of knowledge needed to execute their tasks in the short term and make long-term decisions regarding their employment (Robertson, 2005; Rawlins, 2008). The organizational environment and mechanism of relaying information to employees are the focus of information flow and information adequacy, whereas the content of communication in organizations is the focus of information feedback (Robertson, 2005; Rhee and Moon, 2009). Faculty members and students in educational institutions frequently seek feedback from colleagues and mentorship. Having these points considered, it appears that faculty members may agree with Robertson's (2005) and Rawlins's (2008) notions about information flow within organizations, assuming that organizations should allow the open interchange of information and ideas at work. When faculty encounters high-quality information flow at work, it is expected that they will show evidence of engagement. So, this study proposed the hypothesis about this kind of possible relationship:

*H*_1_*: Information flow plays a role in faculty engagement*.

Furthermore, informational adequacy involves giving faculty members with an appropriate amount of communication on important issues so that they would perform successfully at workplace. Smidts et al. (2001) shared similar viewpoint, claiming that organizational communication among employees consisted of delivering information well about organization and the employee individually. Performing one's everyday work as a faculty member necessitates frequent updates from bosses on personal and individual difficulties (Hartman and McCambridge, 2011). As a result, faculty members' inclination for effective communication shows a need to obtain constant updates on the organization's achievements, objectives, as well as how their specific function is evaluated by the organization. It appears like faculty members who work for organizations that have a lot of knowledge should be more engaged. Similarly, feedback of information would also keep the faculty members engaged to their tasks so the following hypotheses were proposed:

*H*_3_*: information adequacy plays a role in faculty engagement*.

*H*_5_*: information feedback plays a role in student engagement*.

In conclusion, flow of information, adequacy of the information, and information feedback are the three levels of communication that are often discussed inside organizations, because they represent the substance and organizational strategy of employee communication. Authors expect faculty members to be involved with the work if they believe that their organization is backing them and engaging with them. Therefore, this communication must be very pertinent to them.

### Communication Strategies, Engagement, and Organizational Commitment

Faculty engagement, as previously said, is a psychological condition in which faculty members are fully immersed in their work responsibilities and role performance at the workplace. Organizational commitment contrasts with work engagement in that it refers to a person's mindset and attachment to their company, whereas employee engagement focuses on work absorption. Organizations must encourage as well as provide knowledge assets to employees regarding job engagement. It helps them to emerge above the other faculty members (Saks, 2019). In this setting, faculty engagement is the result of an organization's efforts to foster beneficial relationships with its employees.

Furthermore, studies on faculty–organization interactions indicates that providing particular information about faculty status within the organization as well as specific task feedback might improve connections (Karanges et al., 2015; Wang et al., 2019). Organizations' relationship development efforts, in particular, produce a significant amount of commitment. The amount to which other party thinks the interaction is worth investing time and effort to preserve and promote, could be characterized as organizational commitment (Kim et al., 2019). Commitment, in the viewpoint of the faculty, is a long-term desire to stay connected to the company as well as a pleasant feelings disposition toward their workplace. Staff employees receiving assistance from their employer are obliged to put everything into their employment, which causes them to feel more devoted to the company. An urge to continue one's affiliation with an organization is influenced by his work experiences. So, commitment emerges as a result of workplace experiences that are aligned with staff values and meet staff's needs (Albdour and Ikhlas, 2014). Scholars should pay attention to the link between workplace contact with faculty members and organizational commitment. Faculty members frequently request assistance from their superiors and desire unrestricted accessibility to information about their organization's future. According to industry research, educational institution teachers have become less burdened and more likely to desire career security than other professions (Singh and Maini, 2021).

Employees' commitment to their organization is strengthened by open communication, which reduces the possibility that they may explore for other employment prospects beyond their organization (Sadia et al., 2016). This is especially essential for young faculty members. Organizations are significant contributors in improving how individuals of this faculty view their connection with their employers by satisfying the communication demands of young faculty. Work- and job-centered faculty engagement promotes organizational commitment between both academics and staff, according to research (Xie and Derakhshan, 2021). Tomietto et al. (2019) model of job engagement also said that communications and assistance have an impact on employee engagement. Given their workplace communication choices, these challenges of job engagement appear to be particularly pertinent to young faculty. All these arguments suggested the following hypothesis.

*H*_2_*: Information flow plays a role in organizational commitment*.

*H*_4_*: Information adequacy plays a role in organizational commitment*.

*H*_6_*: Information feedback plays a role in organizational commitment*.

*H*_7_*: Faculty engagement plays a role in organizational commitment*.

### Mediating Role of Faculty Engagement

Humanistic approach emphasizes a person's quantifiable, dynamic working, predictable qualities, and emotional capability, rather than workplace negatives such as exhaustion, disagreement, and job discontent. As a result, educational institutions are now looking for faculty who are energetic, dedicated, and focused, i.e., persons who are enthusiastic and engaged with their profession because such faculty members are more efficient and imaginative when they devote their skills and experience to the organization (Minghui et al., 2018). The term “faculty engagement” refers to a pleasant and rewarding emotional state regarding work that is characterized by enthusiasm, determination, and absorption. According to a previous study, the most important aspects of faculty engagement are commitment and vigor (Taris et al., 2017).

Faculty members in educational institutions with greater levels of vigor and passion discover a variety of approaches to address working requirements and barriers while maintaining health and wellbeing. Furthermore, the immersion component, that relates to entire focus on a task, is commonly described by the progression of time or even the trouble of disconnecting himself from someone's activity (Ojo et al., 2021). According to Burić and Macuka (2018), vigor relates to the tangible strength of the mind or body while working; dedication refers to the doer's emotional state, in which he or she is enthusiastic about work; and absorption refers to a cognitive situation in which the individual is completely absorbed in a task.

**Figure 1:**
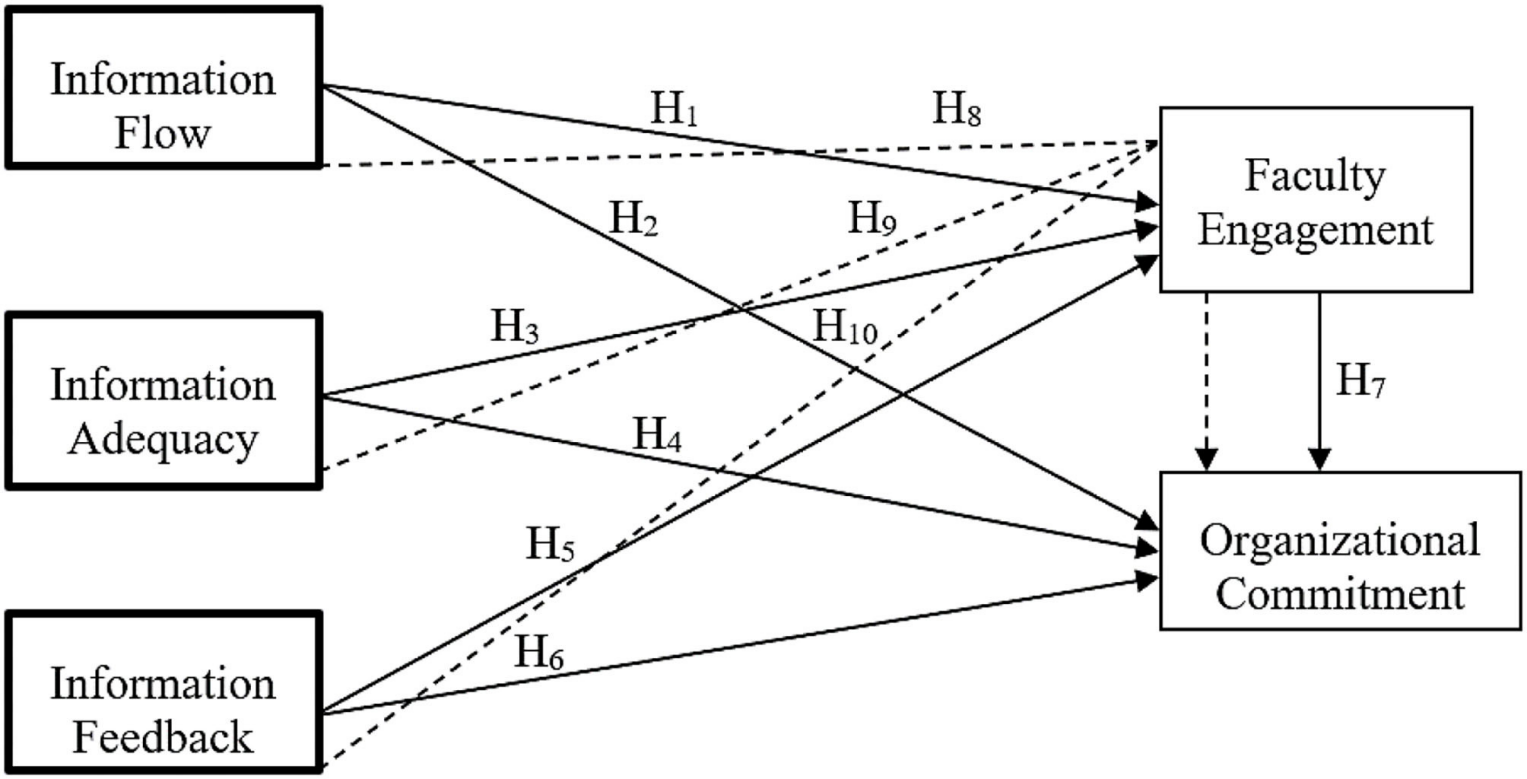
Theoretical framework.

The pleasant and fascinating inspiration which teachers demonstrate in accomplishing their performance targets while feeling totally engaged and committed in performing their job obligations are crucial qualities to clarify job engagement (Wood et al., 2020). Since a prior study demonstrated statistically meaningful and favorable correlations between teacher work engagement and student achievement, teachers' job engagement can be considered crucial in terms of overall school success. Teacher engagement, according to scholars, is one of the indicators of student engagement (Keay, 2018; Butakor et al., 2021). Teachers that are engaged go above and beyond their formal tasks and duties to support their students intellectually using a variety of ways and strategies, resulting in improved academic achievement (Sugianingrat et al., 2019). Hence, the significant role of faculty engagement and the literature discussed in the previous sections, suggested the following role of faculty engagement. Figure 1 shows the bonding of the previous literature and the study module of hypothesis framework.

*H*_8_*: Faculty engagement mediates the relationship of information flow and organizational commitment*.

*H*_9_*: Faculty engagement mediates the relationship of information adequacy and organizational commitment*.

*H*_10_*: Faculty engagement mediates the relationship of information feedback and organizational commitment*.

## Methodology

The hypotheses of the study were developed to analyze the impact of information flow, information adequacy, and information feedback on organizational commitment with the mediation of faculty engagement. The study used a deductive approach and a quantitative research design to test the hypotheses of the study (Nawaz et al., 2022). The hypotheses helped the researcher to investigate the impact of independent variables on dependent variables. The reliability of the study was ensured using a quantitative research design because this design helps to reduce the biases in the study. The questionnaire survey was clear and precise to ensure data rationality. The target population of this study was English language teachers in China. The sample from the entire population was chalked out using the non-probability purposive sampling method. This method saves the resources as the data is collected from readily available respondents (Xiaolong et al., 2021). This particular sector (English language faculty) has been chosen purposefully because it better complements the conceptual model of the study adhering communication strategies with faculty engagement and organizational commitment (Kim and Rhee, 2011). The questionnaires were in English language; therefore, the English language faculty easily and properly understood the questionnaire. A careful estimation of the sample was made based on the study conducted by Wolf et al. (2013) who has investigated the parameter estimate biases in detail along with the scenarios where the parameters affect the statistical power, with a thorough statistical analysis. Wolf et al. (2013) found that a sample size between 30 and 460 cases is found to produce meaningful trends and patterns among the parameters using structural equation modeling. Self-administered survey aided in the data collection process. A total of 350 questionnaires were disbursed among the participants. The respondents were given enough time to fill out the questionnaire. The respondents were told to be natural while filling out the questionnaire and there are no right or wrong responses. A total of 300 questionnaires were collected from the participants over a period of 2 weeks. A total of 24 responses were discarded during the data screening process as they were incomplete or not properly filled by the respondents. A total of 276 responses were found usable after the initial screening for the survey. The usable response rate was 78%. Statistical software was then used for analyzing the data that was obtained from the study participants. The unit of analysis is individuals as the data has been taken from the English language faculty of China.

### Statistical Tool

SmartPLS 3.3.3 software was used as it aids in examine the Structured Equation Modeling (SEM) technique. It is considered as a suitable statistical technique for the analyses based on perceptions (i.e., hypotheses testing). The SEM is an equally helpful technique for all research models irrespective of their nature, i.e., univariate, bivariate, or multivariate. According to Hair et al. (2017), it can be used for both theory exploration and theory confirmation. The partial least square structural equation modeling offers two major advanced regarding data. First of all, traditional multivariate technique requires normal distribution of data, while PLS–SEM is very robust for model estimations and shows flexibility regarding normal distribution, kurtosis and skewness. Second, this technique is also helpful regarding the analysis of ordinal scales along with interval ad ratio scales. This software helps in analyzing the data in a short time with the help of path models such as measurement model and structural model (Nawaz et al., 2020). The recent study has used SmartPLS software for analyzing the data since it is very user friendly and generates detailed results. These models help to determine data reliability, data validity, and significance of the relationships between the constructs. On the basis of *t*-values and *p*-values, the hypotheses are either rejected or accepted.

### Measurement

The data from the study participants were acquired using a 5-point Likert scale. The measurement scales for every construct have been described in detail. The full questionnaire is given in Appendix A.

#### Information Flow

There were four items in the measurement scale of information flow and it was adopted from Rhee and Moon (2009). The sample items are such as “Information in our organization flows openly from the top of the organization downwards.” The Cronbach alpha obtained for this scale is 0.884.

#### Information Adequacy

The measurement scale of information adequacy was adopted from Rhee and Moon (2009) and it consisted of nine items. The sample items are such as “Employees like me receive adequate information how we are being judged from the organization.” The Cronbach alpha obtained for this scale is 0.941.

#### Information Feedback

There were five items on the scale of information feedback and it was adopted from Rosen et al. (2006). The sample items are such as “My supervisor is usually available when I want performance information.” The Cronbach alpha obtained for this scale is 0.922.

#### Faculty Engagement

The measurement scale of faculty engagement was adopted from Schaufeli et al. (2006) and it consisted of nine items. The sample items are such as “At my work, I feel bursting with energy.” The Cronbach alpha obtained for this scale is 0.906.

#### Organizational Commitment

There were five items on the scale of organizational commitment and it was adopted from Gallicano et al. (2012). The sample items are such as “I have a long-lasting bond with the organization I work for.” The Cronbach alpha obtained for this scale is 0.917.

### Demographic Profile

Table 1 below shows the demographic characteristics of the study participants. The table depicts that 57.97% of males and 42.0% of females participated in the study. Moreover, 19.57% of the respondents had an age between 20 and 30 years, 55.47% of the respondents had an age between 31 and 40 years, 16.30% of the respondents had an age between 41 and 50 years, and 14.49% of the respondents had an age above 50 years. The participants had either a bachelor's degree, a master's degree, or a Ph.D., or other degree. A total of 15.58% of the faculty members had a bachelor's degree, 52.27% of the English faculty teacher had a master's degree, while 32.48% had a Ph.D. or other degree. The English faculty teachers who had an experience of <1 year were 18.25%, the English faculty teachers who had an experience of between 1 and 3 years were 43.48%, the English faculty teachers who had an experience of between 4 and 6 years were 27.90%, whereas the English faculty teachers who had an experience of more than 6 years were 10.51%.

**Table 1:** Demographics analysis (*N* = 276).

**Demographics**	**Frequency**	**%**
**Gender**		
Male	160	57.97
Female	116	42.03
**Age (years)**		
20–30	54	19.57
31–40	137	55.47
41–50	45	16.30
Above 50	40	14.49
**Education**		
Bachelors	43	15.58
Masters	144	52.17
Ph.D. and others	89	32.48
**Experience (years)**		
<1	50	18.25
1–3	120	43.48
4–6	77	27.90
More than 6	29	10.51

## Data Analysis and Results

### Measurement Model

**Figure 2:**
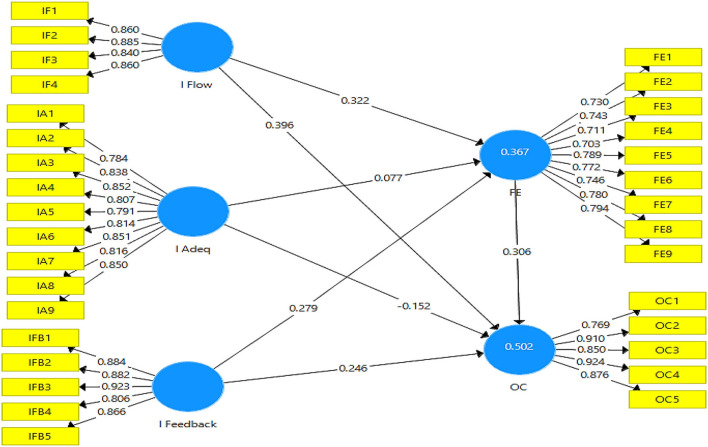
Output of measurement model. IFlow, Information flow; IAdeq, Information adequacy; IFeedback, Information feedback; FE, Faculty engagement; OC, Organizational commitment.

Figure 2 depicts the outer measurement model. The output of the measurement model assists in explaining the contribution of independent variables to outcome variables.

Table 2 demonstrates the model assessment of the direct model. The value of factor loading of more than 0.60 is considered acceptable (Bollen, 2019). The result of the factor loadings for this study shows that the values are above the threshold level; thus, the values are acceptable. The VIF indicator helps in addressing the issue of collinearity. The value of variance inflation factors should be below 5 (Salmerón et al., 2018), and the results revealed that all the values are between 1.657 and 4.938; therefore, the issue of multicollinearity was not detected from the data.

**Table 2:** Model assessment (direct model).

				**Construct reliability and validity**		
	**Factor loadings**		**VIF**	**α**	**Composite reliability**	**AVE**
	IF1	0.860	2.263			
Information flow	IF2	0.885	2.493	0.884	0.920	0.742
	IF3	0.840	2.110			
	IF4	0.860	2.296			
	IA1	0.784	3.008			
Information adequacy	IA2	0.838	4.030			
	IA3	0.852	4.092			
	IA4	0.807	4.298	0.941	0.946	0.677
	IA5	0.791	2.403			
	IA6	0.814	3.219			
	IA7	0.851	4.868			
	IA8	0.816	4.938			
	IA9	0.850	5.181			
	IFB1	0.884	3.851			
	IFB2	0.882	3.500			
Information feedback	IFB3	0.923	3.310	0.922	0.941	0.762
	IFB4	0.806	2.592			
	IFB5	0.866	2.899			
	FE1	0.730	1.865			
Faculty engagement	FE2	0.743	2.596			
	FE3	0.711	2.253			
	FE4	0.703	1.657			
	FE5	0.789	3.341	0.906	0.921	0.566
	FE6	0.772	3.378			
	FE7	0.746	2.795			
	FE8	0.780	4.670			
	FE9	0.794	3.224			
	OC1	0.769	1.665			
Organizational commitment	OC2	0.910	4.082			
	OC3	0.850	2.622	0.917	0.938	0.753
	OC4	0.924	4.888			
	OC5	0.876	3.277			

*IF, Information flow; IA, Information adequacy; IFB, Information feedback; FE, Faculty engagement; OC, Organizational commitment; VIF, Variance inflation factor; α, Cronbach alpha; AVE, Average variance extracted*.

Table 2 also shows the results for construct reliability and validity. According to Taber (2018), for determining the internal consistency the value of Cronbach alpha (α) has to be above 0.70. The table shows that the values are above 0.70. For information flow, the value is (α = 0.884); for information adequacy, the value is (α = 0.941); for information feedback, the value is (α = 0.922); for faculty engagement, the value is (α = *0.906*); and for organizational commitment the value is (α = 0.917); thus, internal consistency exists. Moreover (Benitez et al., 2020), explained that the value of composite reliability must be >0.70 for the data to be reliable. The value obtained for the composite reliability is between 0.920 and 0.950 which indicates that the data came out to be reliable. Furthermore, the average variance extracted (AVE) highlights the presence of convergent validity in the data set. This value must be more than 0.50 (Shrestha, 2021). The value obtained for AVE is between 0.566 and 0.762 which indicates the existence of convergent validity.

The results for Fornell–Larker Criterion and Heterotrait–Monotrait (HTMT) ratio have been shown in Table 3. These tests explain the discriminant validity of the data. As for the Fornell–Larkar criterion, the value at the top column should be higher than the following values on the same column (Nawaz et al., 2019). For example, for faculty engagement, the first value in the column is 0.752, while the following values are 0.476, 0.533, 0.564, and 0.588, which are < 0.752. For the HTMT ratio, the threshold is below 0.90 (Benitez et al., 2020). The results for both these tests are under acceptable ranges; thus, it shows that discriminant validity exists.

**Table 3:** Discriminant validity.

**Constructs**	**FE**	**IA**	**IFB**	**IF**	**OC**
**Fornell–Larcker criterion**					
FE	0.752				
IA	0.476	0.823			
IFB	0.533	0.554	0.873		
IF	0.564	0.756	0.656	0.861	
OC	0.588	0.429	0.585	0.615	0.868
**Heterotrait–Monotrait ratio**					
FE					
IA	0.475				
IFB	0.554	0.589			
IF	0.593	0.819	0.727		
OC	0.621	0.441	0.629	0.677	

*N = 276, IF, Information flow; IA, Information adequacy; IFB, Information feedback; FE, Faculty engagement; OC, Organizational commitment*.

The *R*^2^-value explains the sustainability of the model and its value should be near 0.50 (Akossou and Palm, 2013). The values of *R*^2^ obtained for faculty engagement and organizational commitment are 0.360 and 0.495, respectively. The values are close to 0.50; therefore, the model shows sustainability. The model with values below 5 is said to be free from collinearity (Legate et al., 2021). The table demonstrates that the values are between 1.580 and 3.050; thus, there was no issue of collinearity.

### Structural Model

**Figure 3:**
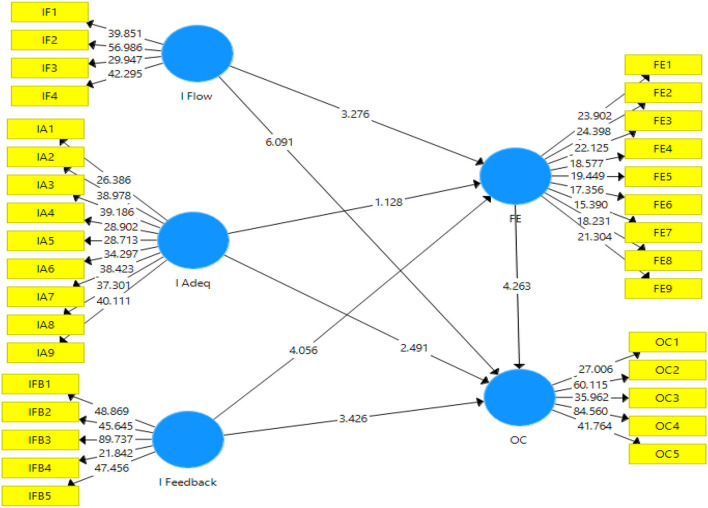
Structural model bootstrapping. IFlow, Information flow; IAdeq, Information adequacy; IFeedback, Information feedback; FE, Faculty engagement; OC, Organizational commitment.

Figure 3 presents the output of structural model bootstrapping. This shows the values of *t*-statistics. The PLS–SEM bootstrapping technique used a 95% confidence interval to accept or reject the proposed hypotheses of this study.

Tables 4, 5 reveal the direct effect and indirect effects of the study. The hypotheses were accepted or rejected on the basis of *p*-values and *t*-statistics. According to Winship and Zhuo (2020), the *t*-statistics have to be more than 1.96. Likewise, the threshold for *p*-value is < 0.50 (95% confidence interval) (Ioannidis, 2018). These tables also present the *f*-values (effect size) that explain the strength of the model. The *f*-values that are close to 1 indicate a strong effect while the values near to 0 signify a weak effect (Funder and Ozer, 2019).

**Table 4:** Direct effects of the variable.

**Paths**	**H**	***O***	***M***	**SD**	***T*-statistics**	***Effect size (f*** **^2^)**	***p***	**Results**
IF → FE	H_1_	0.322	0.315	0.098	3.276	0.057	0.000***	Accepted
IF → OC	H_2_	0.396	0.395	0.065	6.091	0.103	0.001***	Accepted
IA → FE	H_3_	0.077	0.086	0.069	1.128	0.004	0.260	Rejected
IA → OC	H_4_	−0.152	−0.148	0.061	2.491	0.020	0.013*	Accepted
IFB → FE	H_5_	0.279	0.282	0.069	4.056	0.069	0.000***	Accepted
IFB → OC	H_6_	0.246	0.242	0.072	3.426	0.064	0.001***	Accepted
FE → OC	H_7_	0.306	0.306	0.072	4.263	0.119	0.000**	Accepted

N = 276, ^***^p < 0.001, ^**^p < 0.005, ^*^p < 0.05.

*H, Hypothesis; O, Original sample; M, Sample mean; SD, Standard deviation; SRMR = 0.092, NFI = 0.688, IF, Information flow; IA, Information adequacy; IFB, Information feedback; FE, Faculty engagement; OC, Organizational commitment*.

**Table 5:** Indirect effects of the variable.

**Paths**	**H**	**O**	***M***	**SD**	***t*-statistics**	***p***	**Results**
IF → FE → OC	H_8_	0.099	0.097	0.040	2.490	0.013*	Accepted
IA → FE → OC	H_9_	0.024	0.026	0.022	1.058	0.291	Rejected
IFB → FE → OC	H_10_	0.085	0.086	0.028	3.012	0.003**	Accepted

N = 276, ^**^p < 0.005, ^*^p < 0.05.

*H, Hypothesis; O, Original sample; M, Sample mean; SD, Standard deviation; SRMR = 0.092; NFI = 0.688; IF, Information flow; IA, Information adequacy; IFB, Information feedback; FE, Faculty engagement; OC, Organizational commitment*.

Table 4 demonstrates the results for Hypotheses H1–H7, i.e., direct effects of the variables. Hypothesis H1 proposed that information flow plays a role in faculty engagement. The results showed that the acceptance of Hypothesis H1 as (*t* = 3.276; *p* = 0.000). The value of effect size (*f*^2^ = 0.057) indicates the model is weak. Hypothesis H2 proposed that information flow plays a role in organizational commitment. The results showed that the acceptance of Hypothesis H2 as (*t* = 6.091; *p* = 0.001). The value of effect size (*f*^2^ = 0.103) indicates the model is weak. Hypothesis H3 proposed that information adequacy plays a role in faculty engagement. The results showed that the rejection of Hypothesis H3 as (*t* = 1.128; *p* = 0.260). The value of effect size (*f*^2^ = 0.004) indicates the model is very weak. Hypothesis H4 proposed that information adequacy plays a role in organizational commitment. The results showed that the acceptance of H4 as (*t* = 2.492; *p* = 0.000). The value of effect size (*f*^2^ = 0.020) indicates the model is weak. Hypothesis H5 proposed that information feedback plays a role in faculty engagement. The results showed that the acceptance of H5 as (*t* = 4.056; *p* = 0.000). The value of effect size (*f*^2^ = 0.069) indicates the model is weak. Hypothesis H6 proposed that information feedback plays a role in organizational commitment. The results showed that the acceptance of H6 as (*t* = 3.426; *p* = 0.001). The value of effect size (*f*^2^ = 0.064) indicates the model is weak. Hypothesis H7 proposed that faculty engagement plays a role in organizational commitment. The results showed that the acceptance of H7 as (*t* = 4.263; *p* = 0.000). The value of effect size (*f*^2^ = 0.119) indicates the model is weak.

Table 5 presents the results for Hypotheses H8–H10, i.e., the indirect effects of the variables. H8 proposed that faculty engagement mediates the relationship between information flow and organizational commitment. The results showed that the acceptance of Hypothesis H8 as (*t* = 2.490; *p* = 0.013). Hypothesis H9 proposed that faculty engagement mediates the relationship between information adequacy and organizational commitment. The results showed that the rejection of Hypothesis H9 as (*t* = 1.058; *p* = 0.291). Hypothesis H10 proposed that faculty engagement mediates the relationship between information feedback and organizational commitment. The results showed that the acceptance of Hypothesis H10 as (*t* = 3.012; *p* = 0.003).

## Discussion

The purpose of this study was to progress supposition in the field of faculty–organization relationships through analyzing the effect of faculty engagement in these relationships and by gaining a better understanding of how organizations instill committed relationships to faulty members through communication. For this purpose, we created and tested a framework wherein faculty engagement mediated the association between communication strategies and organizational commitment among educational organization's faculty members. The communication strategies included information flow, adequacy of information, and information feedback. The three employee communication variables studied in this study were all drivers of faculty engagement, which was linked to organizational commitment.

This study tried to find out the possible associations between information flow, information adequacy, information feedback, and faculty engagement. This kind of relationships suggested that the teachers, who were communicated properly through the organizational management, were more engaged to their duties. The better the things communicated to the faculty members, better should have been the level of engagement to their jobs. This kind of associations were suggested by some of the scholars of recent past in which the associations of these communication strategies were assessed in terms of job engagement of young generation (Walden et al., 2017). The results showed mixed indications in this study.

The first direct association of information flow with faculty engagement indicated strong association indicating that if information is properly passed on from the administration to the teachers, then it could lead to enhanced dedicated engagement with working by the teachers. The possible reason lies in the flow of information indicating that if information is present but not passed on to the faculty members then it would be difficult for them to do the things on their own. The association of information adequacy with faculty engagement indicated that it was not necessary for the information to be adequate while working of teachers, as there was no significant indication of relationship between both of these. The reason could be drawn from the supposition that the required information for work engagement of teachers does not have any set amount. This information could be of any amount and length as also indicated by Suh et al. (2018).

It was indicated through the results that feedback is very important factor in any communication approach across the organizations (Wang et al., 2020). The results of the association between information feedback and faculty engagement proved that if feedbacks are given properly through communication, then it could lead to more engaged working by the faculty members in educational organizations. The results of all these hypotheses are in accordance with (Walden et al., 2017). They also got the similar results in regard to the millennial generation communication approaches. This study also tried to find out the possible associations of information flow, information adequacy, and information feedback for assessing the organizational commitment of the faculty members. The results indicated a strong positive association between all these three associations.

Similar kind of results were also obtained by Walden et al. (2017) who also found significant associations between communication strategies and the organizational commitment of the employees. The reason behind such results are also alike the reasons for the associations with job engagement of faculty members. The flow of information is necessary, information quantity should be in adequate levels and feedback is also necessary for attaining organizational commitment as were required for the engagement to job. These associations were also indicated by some of the previous researchers in the field of organizational management such as Albdour and Ikhlas (2014) and Sadia et al. (2016). The last direct association was assessed between faculty engagement and organizational commitment.

Previously, a lot of research has been carried out in finding the associations between job engagement, work engagement, student engagement, teachers' engagement, and organizational commitment (Xie and Derakhshan, 2021). These associations were also suggested by Tomietto et al. (2019). The results of this study also indicated that more engaged faculty proves more commitment with their organizations. The mediating or indirect associations were also tested in this study indicating that faculty engagement positively mediated between information flow, information feedback, and organizational commitment. While it could not mediate the relationship between information adequacy and organizational commitment as again the amount of information was not considered necessary component in communication strategies for developing commitment with the organizations. Similar kind of mixed results were also shown by Walden et al. (2017).

### Theoretical Implications

This study has some significant theoretical implications. First, the study examined the role of internal strategies of communication (i.e., information flow, information adequacy, and information feedback) on organizational commitment. Such a model has not been investigated and explored before; therefore this study thoroughly added to the existing public relation and education literature. This study also found that among these three communication strategies, two strategies, i.e., information flow and information feedback significantly impacted the faculty engagement. This study has enriched the literature by examining faculty engagement as a mediator. The reader can understand how significant is faculty engagement in facilitating the relationship between information flow and organizational commitment and between information feedback and organizational commitment. This study would enhance the current knowledge of the readers with regard to communication strategies, organizational commitment, and faculty engagement.

### Practical Implications

This study provides some practical implications that are significant for the management of educational institutes. Based on the results of the result, communication strategies such as information flow, information adequacy, and information feedback are important factors for inducing organizational commitment among the faculty members. Therefore, the management of the institutes should enhance communication strategies by encouraging the employees to collaborate effectively. Hiring supportive leaders who can facilitate the smooth flow of information within the organization. Another way through which communication strategies can be improved is by having a decentralized system within the organization. Moreover, information flow and information feedback are important indicators that help to maximize faculty engagement. Engagement of the faculty can be measured using performance appraisals; hence, the performance appraisals must be conducted quarterly to examine how engaged the faculty members are. Therefore, the role of communication strategies is crucial for improving organizational commitment and faculty engagement.

### Limitations and Recommendations

Although this study was designed to study English language teachers, in the future studies, other subject teachers can also be considered. Western regions and other Asian countries (apart from China) might show different results as the context of the study would be changed in the future studies. Moreover, increasing the sample size would be beneficial in generalizing the data. In the future studies, a mixed approach can be deployed to have an in-depth understanding of the relationship between study variables. This study examined the role of three different communication strategies, i.e., information flow, information adequacy, and information feedback on organizational commitment with the mediation of faculty engagement. The future studies can examine the role of faculty motivation as a mediator and organizational support as a moderator in the relationship between communication strategies and organizational commitment.

## Conclusion

Fostering organizational commitment for the employees is necessary for the organization to deliver high-quality service to the end-user. In this regard, organizations are seeking ways to enhance organizational commitment through communication strategies. Therefore, this study investigated the role of communication strategies (i.e., information flow, information adequacy, and information feedback) on organizational commitment with the mediation of faculty engagement among English language teachers in China. The outcome of the study demonstrated that information flow and information feedback significantly impact organizational commitment and faculty engagement. The analysis also revealed that information adequacy significantly impacts organizational commitment but has no relationship with faculty engagement. The mediation analysis demonstrated that faculty engagement mediated the relationship between information flow and organizational commitment; and between information feedback and organization commitment. However, faculty engagement did not mediate the relationship between information adequacy and organizational commitment among English language teachers in China.

## Data Availability Statement

The original contributions presented in the study are included in the article/supplementary material, further inquiries can be directed to the corresponding author/s.

## Ethics Statement

The studies involving human participants were reviewed and approved by Lanzhou City University, China. The participants provided their written informed consent to participate in this study. The study was conducted in accordance with the Declaration of Helsinki.

## Author Contributions

YM conceived, designed the concept, collected the data, wrote the article, read, and agreed to the published version of the manuscript.

## Conflict of Interest

The author declares that the research was conducted in the absence of any commercial or financial relationships that could be construed as a potential conflict of interest.

## Publisher's Note

All claims expressed in this article are solely those of the authors and do not necessarily represent those of their affiliated organizations, or those of the publisher, the editors and the reviewers. Any product that may be evaluated in this article, or claim that may be made by its manufacturer, is not guaranteed or endorsed by the publisher.

## Appendix A (Questionnaire)

*Information flow*

1. Information in our organization flows openly from the top of the organization downward.
2. Information in our organization flows openly to the top of the organization.
3. Information in our organization flows openly between workgroups/departments.
4. Information in our organization flows openly throughout the overall organization.

*Information adequacy*

1. Employees like me receive adequate information how we are being judged from the organization.
2. Employees like me receive adequate job performance information from the organization.
3. Employees like me receive adequate information about employee welfare from our organization.
4. Employees like me receive adequate information about our progress in our job from the organization.
5. Employees like me receive adequate information about the goals of the organization.
6. Employees like me receive adequate information about changes within our organization.
7. Employees like me receive adequate information about policy within the organization.
8. Employees like me receive adequate information about accomplishments of the organization.
9. Employees like me receive adequate information how organization profits and standing.

*Faculty engagement*

1. At my work, I feel bursting with energy.
2. At my job, I feel strong and vigorous.
3. I am enthusiastic about my job.
4. My job inspires me.
5. When I get up in the morning, I feel like going to work.
6. I feel happy when I am working intensely.
7. I am proud of the work that I do.
8. I am immersed in my work.
9. I get carried away when I am working.

*Organizational commitment*

1. I have a long-lasting bond with the organization I work for.
2. Compared to other potential employers, I value my relationship with the organization I work for more.
3. I would rather work together with this organization than not.
4. I feel that the organization I work for is trying to maintain a long-term commitment to me.
5. I can see that the organization I work for wants to maintain a relationship with me.

*Information feedback*

1. My supervisor is usually available when I want performance information.
2. My supervisor is too busy to give me feedback.
3. I have little contact with my supervisor.
4. I interact with my supervisor on a daily basis.
5. The only time I receive performance feedback from my supervisor is during my performance review.
